# An EGFP-marked recombinant lactobacillus oral tetravalent vaccine constitutively expressing α, ϵ, β1, and β2 toxoids for *Clostridium perfringens* elicits effective anti-toxins protective immunity

**DOI:** 10.1080/21505594.2019.1653720

**Published:** 2019-08-20

**Authors:** Guojie Ding, Jing Bai, Baohua Feng, Li Wang, Xinyuan Qiao, Han Zhou, Yanping Jiang, Wen Cui, Lijie Tang, Yijing Li, Yigang Xu

**Affiliations:** a Heilongjiang Key Laboratory for Animal Disease Control and Pharmaceutical Development, College of Veterinary Medicine, Northeast Agricultural University, Harbin, P.R. China; b Northeast Science Inspection Station, Key Laboratory of Animal Pathogen Biology of Ministry of Agriculture of China, Northeast Agricultural University, Harbin, P.R. China

**Keywords:** Clostridium perfringens, toxoids, lactobacillus, enhanced green fluorescent protein, oral immunization

## Abstract

*Clostridium perfringens* is a common opportunistic pathogen endangering livestock and poultry breeds. Here, using enhanced green fluorescent protein as screening marker, a recombinant lactobacillus tetravalent vaccine constitutively expressing α, ϵ, β1, and β2 toxoids of *C. perfringens* was developed, and its immunogenicity in mice was investigated via oral administration. This probiotic vaccine could effectively induce antigen-specific secretory IgA (sIgA)-based mucosal and IgG-based humoral immune responses, and significantly high levels (*p*< 0.05) of cytokines IL-2, IL-4, IL-10, IL-12, IL-17, and IFN-γ were produced in immunized mice. Moreover, lymphoproliferation and percentage of CD4^+^ and CD8^+^ T cells significantly increased in mice of the probiotic vaccine group. Challenge experiments were performed in mice with *C. perfringens* toxinotypes A, C, and D crude toxins to evaluate protection efficiency of the probiotic vaccine, using a commercial inactivated *C. perfringens* vaccine made by *C. perfringens* toxinotypes A, C, and D as vaccine control. We observed 80% protection rate in the probiotic vaccine group, which was higher than commercial vaccine group, whereas all mice in control groups died and obvious histopathological changes were observed in liver, spleen, kidney, and intestines of mice. Significantly, we compared the immunogenicity and protection efficiency of lactobacillus constitutive expression system and lactobacillus inducible expression system, and results showed that lactobacillus constitutive expression system has obvious advantages. Our study clearly demonstrated that the probiotics vaccine could effectively induce mucosal, humoral, and cellular immunity, and provide effective protection against *C. perfringens* toxins, suggesting a promising strategy for the development of oral vaccine against *C. perfringens*.

## Introduction

*Clostridium perfringens* (*C. perfringens*), a Gram-positive, spore-forming anaerobic bacillus that is widely distributed in natural environments and can usually be found in the gastrointestinal tract of livestock and poultry as an opportunistic pathogen, is associated with human food-borne diseases as well as animal diseases [1,2], and exotoxins are its main virulence factors. According to the traditional *C. perfringens* toxin typing scheme, *C. perfringens* is classified into five toxin types (A, B, C, D, and E) based on the major toxins α, β, ϵ, and ι produced by each toxinotype [3], while a recently updated study proposed that *C. perfringens* strains should be classified into seven toxinotypes, A to G [4]. The α-toxin with phospholipase C and sphingomyelinase activities is a major pathogenic factor influencing *C. perfringens* infections that are responsible for gas gangrene in sheep [5], and humans [6], which can be produced by all toxinotypes of *C. perfringens* but is the only toxin produced by toxinotype A [7,8]. The β-toxin (β1-toxin) produced by toxinotype B and C is a key virulence factor of fatal hemorrhagic enterocolitis and enterotoxemia in livestock and can lead to severe intestinal bleeding and small intestinal mucosal necrosis [9,10]. The *C. perfringens* β2-toxin produced by newly characterized β2-toxigenic *C. perfringens* shows a low homology to β1-toxin, and is involved in enterocolitis [11,12]. The ϵ-toxin is a potent pore-forming toxin responsible for central nervous system disease in ruminant animals followed by blood-brain barrier dysfunction and white matter injury [13]. *C. perfringens* can cause various diseases in different animals, including gas gangrene, acute enterotoxemia, and enteritis syndrome. Therefore, the development of an effective vaccine against *C. perfringens* toxins is of great importance.

Currently, although in-feed antibiotic, such as virginiamycin and tylosin, is the most effective approach for the control of *C. perfringens* infections in livestock and poultry, antibiotics can result in many negative effects on the environment as well as human health [14]. Parenteral vaccination with inactivated vaccines/subunit vaccines is usually effective in eliciting systemic immune responses, which could provide effective protection against many animal clostridial diseases [15]. However, parenteral vaccination could not induce secretory IgA (sIgA)-based mucosal immunity, because *C. perfringens* is an opportunistic pathogen in the gastrointestinal tract of animals and its enterotoxins are absorbed via intestinal mucosa. Therefore, effective oral vaccines that can induce a more efficacious sIgA-based protective mucosal immunity are urgently needed for the prevention of *C. perfringens* diseases. An ideal oral vaccine would not only effectively deliver antigenic matter to the immune system to induce protective mucosal and systemic immune responses, but also the delivery vehicle itself should be safe and harmless.

*Lactobacillus* is a Gram-positive, facultative anaerobic or microaerophilic, rod-shaped, non-spore-forming bacteria, which is the most common probiotic found in food and the intestinal tract of humans and animals [16]. Studies have shown that some specific isolates of lactobacilli have potential immunomodulatory properties, such as promoting strong adhesive interactions with intestinal epithelial cells [17] and preventing injury of the epithelial cell barrier [18], anti-inflammatory capacity[19], modulating innate immune response [20] and sIgA production[21], regulating immunological functions of dendritic cells, and T helper cells activation [22,23]. Moreover, lactobacilli can survive transit through the upper gastrointestinal tract and colonize the intestinal tract [24,25]. Therefore, lactobacilli are widely used as an oral vaccine delivery vehicle to express heterologous antigens, thereby providing effective immunogenicity via oral administration [26–32].

In the present study, using enhanced green fluorescent protein (EGFP) as a screening marker for recombinant lactobacillus and *C. perfringens* α, ϵ, β1, and β2 toxoids as antigens, a genetically engineered *Lactobacillus casei* 393 (*L. casei* 393) strain pPG-Δ-E-α-β2-ϵ-β1/*L. casei* 393 with non-antibiotic resistance constitutively expressing toxoids of *C. perfringens* α, ϵ, β1, and β2 toxins were successfully constructed with a lactobacillus constitutive expression plasmid pPG-T7g10-PPT constructed in our lab, and its immunogenicity and immune protective efficacy were evaluated in BALB/c mice via oral vaccination, and subjected to comparison with the lactobacillus inducible expression system pPG-2-α-β2-ϵ-β1/*L. casei* 393 with chloromycetin resistance constructed previously by our lab [33].

## Materials and methods

### Strains, plasmids, and animals

**Figure 1.**
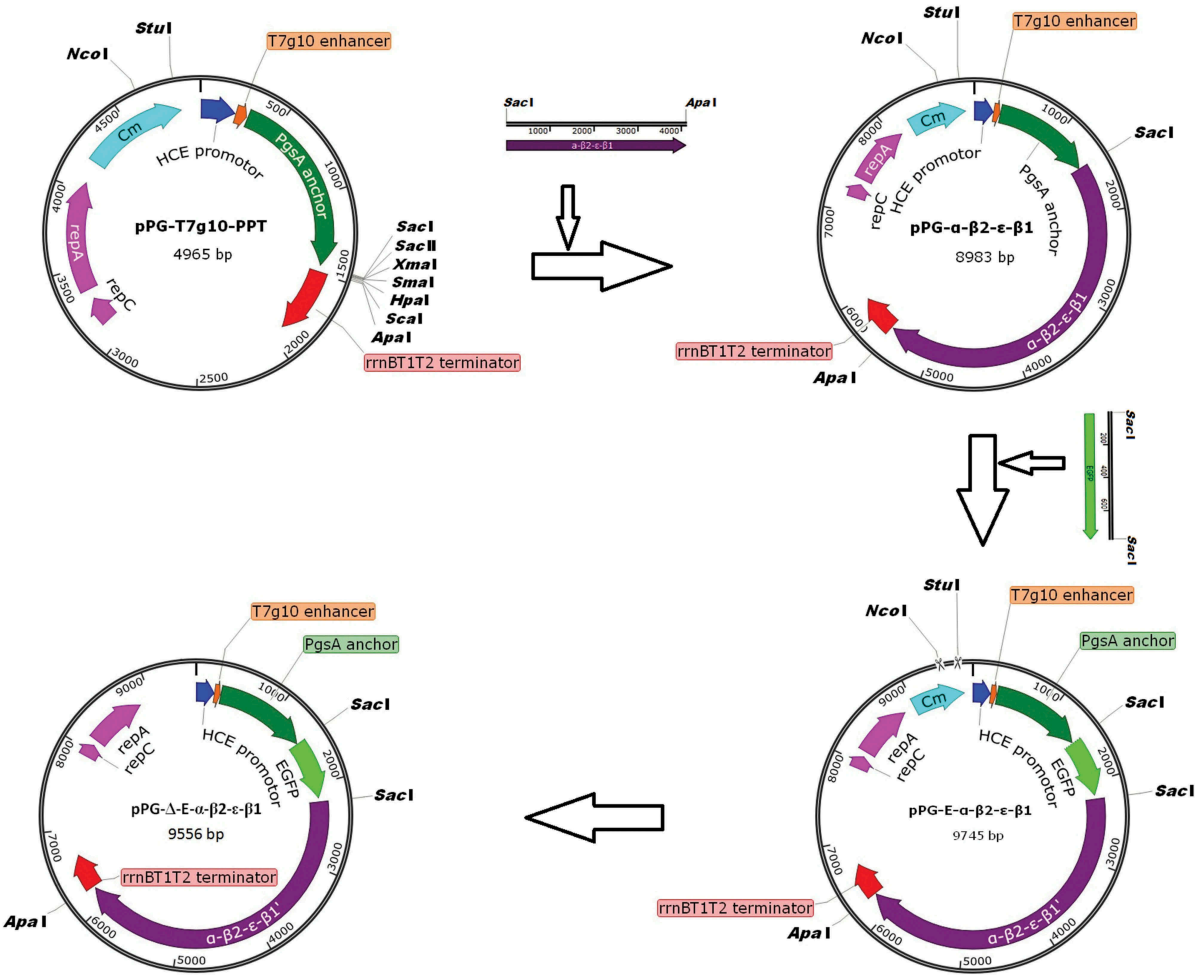
Construction process of the recombinant pPG-Δ-E-α-β2-ϵ-β1 plasmid. The constitutive expression plasmid pPG-T7g10-PPT contains a HCE strong constitutive promoter, a T7g10 transcriptional enhancer, a PgsA anchor from *Bacillus subtilis*, and an rrnBT1T2 terminator.

*C. perfringens* toxinotype A (C57-1), toxinotype C (CACC-61), and toxinotype D (CCVC-81) were purchased from the China Institute of Veterinary Drug Control (Beijing, China). *L. casei* ATCC393 was grown anaerobically in de Man, Rogosa and Sharpe (MRS) broth (Sigma, USA) at 37°C without shaking. The plasmid pMD19-T-EGFP containing the gene encoding EGFP (GenBank accession no. U57607) and the plasmid pMD18-T-α-β2-ϵ-β1 containing the fusion genes encoding toxoids of *C. perfringens* α (H68G), β2 (C234G), ϵ (H106P), and β1 (D81A, K83A, and C292A) was constructed by our laboratory [30,33]. The constitutive expression plasmid pPG-T7g10-PPT (as shown in Figure 1) containing a HCE strong constitutive promoter cloned from the d-amino acid aminotransferase, a T7g10 transcriptional enhancer, a PgsA anchor from *Bacillus subtilis* for surface-displaying heterologous protein on the cell membrane, and an rrnBT1T2 terminator was constructed by our laboratory [30,31]. A xylose-inducible expression recombinant lactobacillus pPG-2-α-β2-ϵ-β1/*L. casei* 393 with Chloromycetin resistance was constructed previously by our lab [33]. Four-week-old specific pathogen-free (SPF) BALB/c mice purchased from Liaoning Changsheng Biotechnology Company in Liaoning, China were kept under SPF conditions with free access to standard water and diet. This study was carried out in accordance with the recommendations in the Guide for the Care and Use of Laboratory Animals of the National Institutes of Health. The protocol was approved by the Ethical Committee for Animal Experiments of Northeast Agricultural University, China.

### *Construction of recombinant* lactobacillus *strain*

Recombinant expression plasmids were constructed as shown in Figure 1. Briefly, the fusion genes in pMD18-T-α-β2-ϵ-β1 encoding *C. perfringens* α, β2, ϵ, and β1 toxoids were subcloned into the constitutive expression vector pPG-T7g10-PPT, giving rise to pPG-α-β2-ϵ-β1. Next, the gene encoding the EGFP was inserted upstream of the fusion gene α-β2-ϵ-β1 and linked with α-β2-ϵ-β1 by a linker, generating the plasmid pPG-E-α-β2-ϵ-β1. Subsequently, the chloramphenicol-resistant gene in pPG-E-α-β2-ϵ-β1 was deleted by enzymatic digestion with *Nco*I and *Stu*I followed by ligation of the blunt-ended DNA, giving rise to recombinant pPG-Δ-E-α-β2-ϵ-β1 with EGFP marker. Finally, the recombinant pPG-Δ-E-α-β2-ϵ-β1 was electroporated into *L. casei* 393 competent cells, followed by screening of the positive recombinants by flow cytometry. Briefly, the strain pPG-Δ-E-α-β2-ϵ-β1/*L. casei* 393 was grown in MRS medium at 37°C for 16 h without shaking, followed by centrifuging and washing with sterile PBS, the bacteria were subjected to screening by flow cytometry, and the bacterial strain with green fluorescent signal was separated, and cultured in 96-well cell plate containing MRS agar medium at 37°C. Overall, the EGFP-marked recombinant lactobacillus strain pPG-Δ-E-α-β2-ϵ-β1/*L. casei* 393 was successfully constructed.

### Protein expression

For identification of EGFP expression, the recombinant pPG-Δ-E-α-β2-ϵ-β1/*L. casei* 393 was grown in normal MRS medium at 37°C for 16 h without shaking, and then the bacterial culture was centrifuged at 10,000 × g for 5 min followed by washing with sterile PBS for three times, resuspended in PBS, and then the expression of EGFP was analyzed by ultra-high resolution microscopy and flow cytometry. For identification of constitutive expression of *C. perfringens* α, β2, ϵ, and β1 toxoids, the pPG-Δ-E-α-β2-ϵ-β1/*L. casei* 393 was cultured overnight in basic MRS medium supplemented with raffinose, sucrose, lactose, trehalose, glucose, and fructose as carbon sources, respectively. Then, the bacterial cultures were centrifuged at 10,000 × g for 5 min, and the cell pellets were washed twice with sterile 25 mmol/L Tris-Cl (pH8.0) and lysed with 2× SDS buffer solution followed by boiling for 10 min. After centrifugation at 12,000 × g for 10 min, the supernatant was subjected to 10% sodium dodecyl sulfate-polyacrylamide gel electrophoresis (SDS-PAGE), and the proteins were then electrotransferred onto a PVDF membrane, followed by immunoblots developed with mouse anti-α/β2/ϵ/β1 toxoid monoclonal antibody (diluted at 1:500) as the primary antibody prepared by our lab followed by horseradish peroxidase (HRP)-conjugated goat anti-mouse IgG (Sigma, USA) diluted 1:2000 as the secondary antibody. The immunolabelled bands were visualized using the chemiluminescent substrate reagent (Pierce, USA) according to the manufacturer’s instruction.

### Colonization ability of recombinant strain in the intestinal tract of mice

The recombinant pPG-Δ-E-α-β2-ϵ-β1/*L. casei* 393 was cultured to an OD_600_ of 1.0 and then centrifuged at 5000 × g for 10 min followed by washing twice with sterile PBS. The bacteria pellets were then resuspended to a concentration of 10^10^ CFU/mL. Next, the recombinant lactobacilli were labeled with 5ʹ (and 6ʹ)-carboxyfluorescein diacetate succinimidyl ester (CFDA-SE) at 37°C for 25 min, and then washed twice with sterile PBS to remove excess CFDA-SE. Finally, the recombinant lactobacilli were resuspended in sterile PBS, and the labeling rate was detected by flow cytometry. A total of 24 mice were orally administrated with approximately 10^9^ CFU/mL of the CFDA-SE-labeled recombinant lactobacilli per mice, and groups of three mice each were sacrificed on days 1, 3, 5, 7, 9, 11, 13, and 15, and then the duodenum, jejunum, ileum, and colon were extracted and cut longitudinally, and visible residual food particles or fecal material was removed from individual sections prior to the detection of adhering CFDA-SE-labeled lactobacilli. After dislodging microbes from the mucosal surfaces of each intestinal section with PBS and being fixed with 0.75% formaldehyde (v/v) as previously described [34], flow cytometric analysis was performed using the microbes extracted from the mice fed orally with sterile PBS as control.

### Immunization

Prior to oral immunization, recombinant pPG-Δ-E-α-β2-ϵ-β1/*L. casei* 393 was adjusted to a concentration of 1 × 10^10^ CFU/mL. BALB/c mice were randomly divided into five groups. Mice in the probiotic vaccine group (n = 64) were orally immunized with 100 μL of recombinant pPG-Δ-E-α-β2-ϵ-β1/*L. casei* 393. Mice in the control group (n = 64) were immunized with an equivalent dose of pPG-T7g10-PPT/*L. casei* 393. Mice in the mock control group (n = 64) received 100 μL of PBS. Mice in the recombinant lactobacillus control group (n = 64) were immunized with an equivalent dose of pPG-2-α-β2-ϵ-β1/*L. casei* 393. Mice in the vaccine control group (n = 40) were injected intramuscularly with commercial inactivated *C. perfringens* vaccines produced for sheep that were made by *C. perfringens* toxinotypes A, C, and D (China Animal Husbandry Industry, Beijing, China) according to the manufacturer’s instruction. Oral immunization protocol was performed on three consecutive days (days 0, 1, and 2), and a booster immunization was given at days 14, 15, and 16, and a second booster was given at days 28, 29 and 30 [26].

### ELISA

Sera, intestinal mucus, and fecal samples were prepared on days 0, 7, 14, 21, 28, 35, and 42 after immunization and used for detecting specific IgA or IgG antibodies by ELISA. Briefly, polystyrene microtiter plates were, respectively, coated overnight at 4°C with 10 μg of *C. perfringens* α, β2, ϵ, and β1 toxoid proteins that were expressed by *E. coli* and purified in our lab. Next, the plates were washed thrice with PBS containing 1% Tween-20, and then saturated with PBS containing 5% skimmed milk at 37°C for 2 h. The sera, intestinal mucus, and fecal extraction were used as the primary antibody, and horseradish peroxidase-conjugated goat anti-mouse IgA or IgG (Sigma, USA) was used as a secondary antibody, and followed by color development using tetramethylbenzidine (TMB) (TIANGEN, China) as the substrate, and then absorbance was measured at 450 nm. Moreover, the levels of cytokines IL-4, IL-12, IL-10, IL-17, IL-2, and IFN-γ in the sera samples were detected by ELISA Kits according to the manufacturer’s instructions (Biosource International, USA).

### Detection of CD3^+^, CD4^+^, and CD8^+^ t cells by flow cytometric analysis

Seven days after a second booster immunization with pPG-Δ-E-α-β2-ϵ-β1/*L. casei* 393 administered on days 28, 29, and 30, spleen lymphocytes were collected from the mice in the vaccine and control groups, and were adjusted to 2 × 10^6^/mL. Then, the lymphocytes were stained with FITC anti-mouse CD4, APC anti-mouse CD3, and PE anti-mouse CD8 fluorescence-labeled antibody (Sigma, USA) at room temperature for 30 min, respectively. After washing thrice with PBS, flow cytometry was performed using a FACS Caliber (Becton Dickinson, USA) to determine the T cell subsets.

### Proliferation of lymphocytes

On day 42 after the primary immunization, the immunized mice were euthanized and spleen cells were obtained for a lymphocyte proliferation test. In brief, 100 μL of spleen cell suspensions (5 × 10^6^ cells/mL) in eight duplicates were incubated in a 96-well plate containing RPMI 1640 medium plus 10% fetal bovine serum at 37°C in a 5% CO2 incubator for 8–12 h. The cells were then restimulated with 0.01 mg/mL, 0.1 mg/mL, 1.0 mg/mL, and 10 mg/mL of purified recombinant α-β2-ϵ-β1 protein for 60 h, respectively (culture medium used as blank control). Next, 10 μL of thiazolyl blue tetrazolium bromide (MTT) at 5 mg/mL was added into each well and continually incubated at 37°C for 4 h. Lymphocyte proliferation was assessed by the CellTiter 96 AQueous Non-Radioactive Cell Proliferation Assay according to the manufacturer’s instructions (Promega, USA), followed by absorbance measured at 600 nm. The stimulation index was calculated as follows: SI = OD_600 (sample)_/OD_600 (blank control)_.

### Challenge tests

To evaluate the protective efficacy of recombinant pPG-Δ-E-α-β2-ϵ-β1/*L. casei* 393 via oral immunization, challenge tests were performed. On day 7 after the second booster immunization, each group of mice was randomly divided into two subgroups (20 mice per subgroup). The mice in one subgroup were intramuscularly injected with 1× LD_100_ (60 μg _toxin_/g _body weight_) of the *C. perfringens* toxinotype A, C, and D crude toxin mixture. The mice in another subgroup were orally challenged with 200 μL of the *C. perfringens* toxinotype A, C, and D mixture (1 × 10^10^ CFU/mL). The daily survival rate of the mice in each group was recorded post-challenge, and the histopathological changes in the liver, kidney, heart, colon, spleen, and brain of the mice immunized with recombinant pPG-Δ-E-α-β2-ϵ-β1/*L. casei* 393, commercially available inactivated *C. perfringens* vaccine, pPG-T7g10-PPT/*L. casei* 393, and PBS were observed, respectively.

### Statistic analysis

The data were presented as mean ± standard deviation (SD). Statistical significance was determined using SPSS software (IBM) for ANOVA, and expressed as the following *p* values: *p* < 0.05 (*) and *p* < 0.01 (**).

## Results

### Constitutive expression of the fusion protein

**Figure 2.**
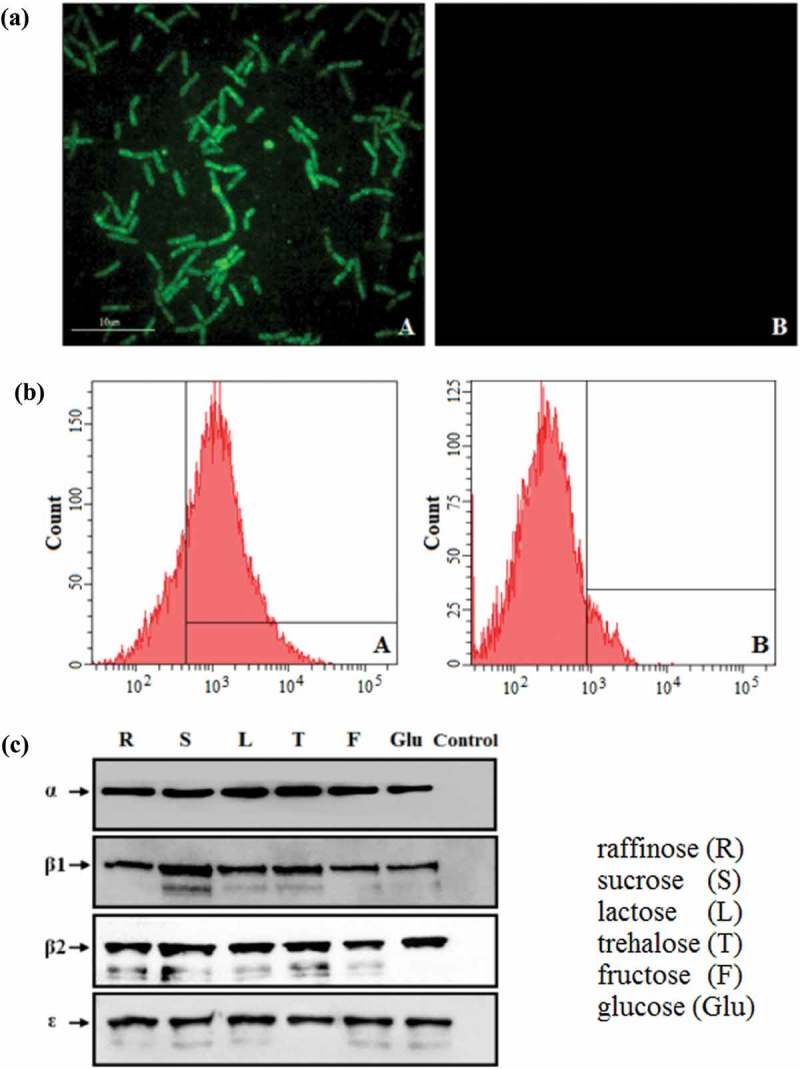
Constitutive expression of proteins. (a) The pPG-Δ-E-α-β2-ϵ-β1/*L. casei* 393 cultured in normal MRS broth was analyzed by ultra-high-resolution microscopy, and results showed that significant green fluorescence was observed (Figure 2(a): A), but not in the pPG-T7g10-PPT/*L. casei* 393 strain (Figure 2(a): B). (b) Flow cytometry results showed that EGFP was effectively expressed by pPG-Δ-E-α-β2-ϵ-β1/*L. casei* 393 (Figure 2(b): A). (c) Western blot results showed that the α-β2-ϵ-β1 fusion protein can be constitutively expressed by the pPG-Δ-E-α-β2-ϵ-β1/*L. casei* 393 strain using raffinose (R), sucrose (S), lactose (L), trehalose (T), glucose (Glu), and fructose (F) as carbon sources, respectively.

The expression of the EGFP screening marker by pPG-Δ-E-α-β2-ϵ-β1/*L. casei* 393 cultured in normal MRS medium was identified by ultra-high resolution microscopy and flow cytometric analysis, and the results showed that significant green fluorescence was observed on the pPG-Δ-E-α-β2-ϵ-β1/*L. casei* 393 (Figure 2(a): A), but not on the pPG-T7g10-PPT/*L. casei* 393 (Figure 2(a): B). Flow cytometric analysis also showed that the EGFP marker was effectively expressed by the recombinant lactobacilli (Figure 2(b): A). In order to confirm the constitutive expression of the fusion protein, the pPG-Δ-E-α-β2-ϵ-β1/*L. casei* 393 was grown in basic MRS broth supplemented with different carbon sources, and the immunoblot results showed that the α-β2-ϵ-β1 fusion protein of *C. perfringens* was constitutively expressed by the pPG-Δ-E-α-β2-ϵ-β1/*L. casei* 393 (Figure 2C), which can be recognized by mouse anti-α, β2, ϵ, and β1 toxoid monoclonal antibody, respectively.

### Colonization of recombinant lactobacilli in mice

Recombinant pPG-Δ-E-α-β2-ϵ-β1/*L. casei* 393 was labeled with CFDA-SE (labeling rate reached 99.7%, data not shown) and its colonization ability in the intestinal tracts was measured via oral administration of CFDA-SE-labeled lactobacilli to mice. Flow cytometric analysis showed that the pPG-Δ-E-α-β2-ϵ-β1/*L. casei* 393 could survive and adhere to the duodenum, jejunum, ileum, and colon of mice (Table 1). On day 1 after the oral administration, the positive rate of CFDA-SE-labeled lactobacilli in the duodenum, jejunum, ileum, and colon was 65.9%, 86.7%, 67.2%, and 78.2%, respectively. With the metabolism of mice and the influence of the digestive tract environments, the colonization rate of recombinant lactobacilli was gradually decreased. By day 15, the positive rate of recombinant lactobacilli still remained 10.3%, 19.5%, 13.8%, and 15.2% in the duodenum, jejunum, ileum, and colon, respectively.

**Table 1.** The percentage of CFDA-SE-labeled recombinant lactobacilli in the different intestinal sections of mice after orogastric intubation.

Days	% of population (10^4^)			
	Duodenum	Jejunum	Ileum	Colon
1	65.9 ± 2.1	86.7 ± 4.1	67.2 ± 8.5	78.2 ± 2.5
3	48.2 ± 2.1	76.9 ± 1.4	58.7 ± 2.3	65.8 ± 3.2
5	43.3 ± 5.3	71.8 ± 1.8	52.0 ± 3.5	55.6 ± 3.5
7	34.4 ± 3.7	69.3 ± 0.6	41.0 ± 6.1	44.0 ± 5.9
9	28.6 ± 1.7	57.9 ± 2.1	33.0 ± 2.0	37.0 ± 3.6
11	21.3 ± 2.1	50.1 ± 1.6	27.5 ± 1.3	32.3 ± 0.8
13	16.1 ± 7.5	37.4 ± 4.5	20.3 ± 4.5	26.5 ± 1.0
15	10.3 ± 2.5	19.5 ± 3.3	13.8 ± 2.6	15.2 ± 0.6

### Determination of specific antibody

**Figure 3.**
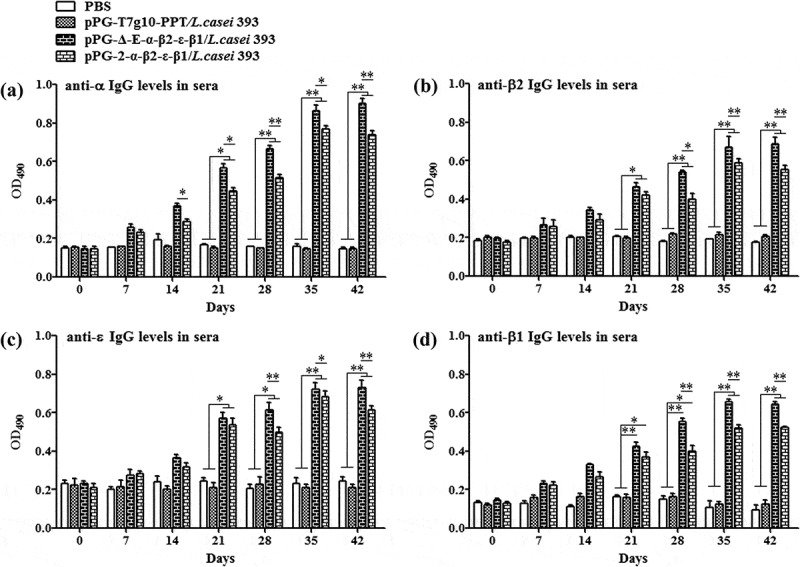
Antigen-specific IgG antibodies in sera. Sera samples collected from mice orally immunized with pPG-Δ-E-α-β2-ϵ-β1/*L. casei* 393, pPG-2-α-β2-ϵ-β1/*L. casei* 393, pPG-T7g10-PPT/*L. casei* 393, and PBS were determined for levels of anti-α specific IgG (a), anti-β2 specific IgG (b), anti-ϵ specific IgG (c), and anti-β1 specific IgG (d) by ELISA (*p < 0.05, **p < 0.01). The results are presented as mean ± SD in each group.

**Figure 4.**
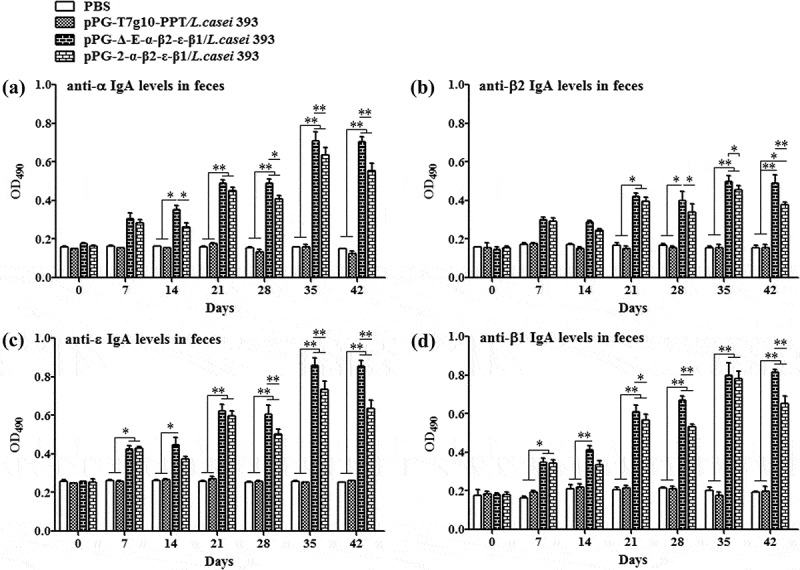
Antigen-specific IgA antibodies in feces. Fecal samples were collected from each group on days 0, 7, 14, 21, 28, 35, and 42 after the primary immunization, and the levels of anti-α specific IgA (a), anti-β2 specific IgA (b), anti-ϵ specific IgA (c), and anti-β1 specific IgA (c) were determined by ELISA (*p < 0.05, **p < 0.01). The results are presented as mean ± SD of each group.

**Figure 5.**
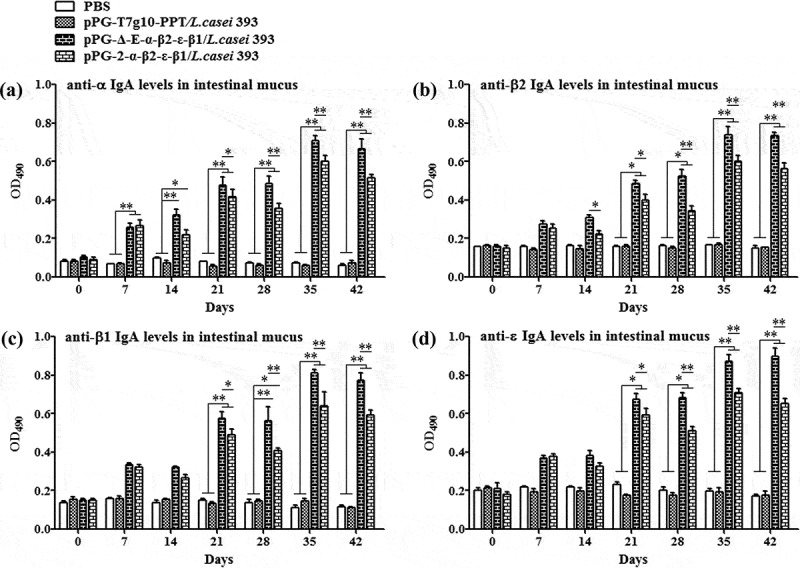
Antigen-specific IgA antibodies in intestinal mucus. Intestinal mucus samples collected from each group on days 0, 7, 14, 21, 28, 35, and 42 after the primary immunization, and levels of anti-α specific IgA (a), anti-β2 specific IgA (b), anti-ϵ specific IgA (c), and anti-β1 specific IgA (c) were determined by ELISA (*p < 0.05, **p < 0.01). The results are presented as mean ± SD of each group.

**Figure 6.**
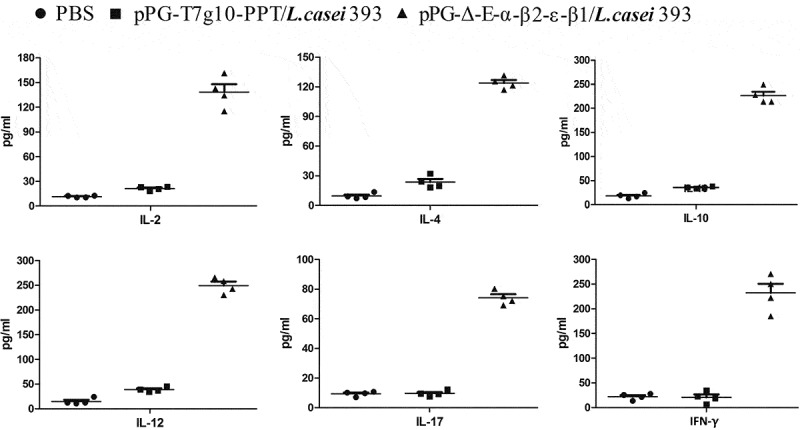
The levels of cytokines IL-2, IL-4, IL-10, IL-12, IL-17, and IFN-γ in sera samples determined by ELISA. The results are presented as mean ± SD of each group.

The ability of recombinant pPG-Δ-E-α-β2-ϵ-β1/*L. casei* 393 to induce mucosal and systemic immune responses was evaluated by detecting the presence of specific anti-α/β2/ϵ/β1 toxoid IgA and IgG antibodies by ELISA, respectively. The results showed that the pPG-Δ-E-α-β2-ϵ-β1/*L. casei* 393 could effectively induce mucosal and systemic immune responses, and significant levels (*p*< 0.01) of anti-α/β2/ϵ/β1 toxoid protein-specific systemic IgG (Figure 3) and mucosal sIgA in feces (Figure 4) and intestinal mucus (Figure 5) were elicited in the mice orally immunized with recombinant pPG-Δ-E-α-β2-ϵ-β1/*L. casei* 393 after the second booster, indicating a better immunogenicity. However, there was no difference (*p*> 0.05) observed in the mice of pPG-T7g10-PPT/*L. casei* 393 group and PBS group before and after immunization. By contrast, the IgG and IgA antibody levels in the mice induced by the constitutive recombinant pPG-Δ-E-α-β2-ϵ-β1/*L. casei* 393 were significantly higher (*p*< 0.01) than that induced by the xylose-inducible expression recombinant pPG-2-α-β2-ϵ-β1/*L. casei* 393 after the second booster. Furthermore, the levels of cytokines IL-4, IL-12, IL-10, IL-17, IL-2, and IFN-γ in the sera samples collected on days 35 after the primary immunization were determined by ELISA kits, and the results showed that significant levels of cytokines were detected in the sera of the mice orally immunized with pPG-Δ-E-α-β2-ϵ-β1/*L. casei* 393 (Figure 6), compared with the pPG-T7g10-PPT/*L. casei* 393 or PBS group.

### CD4^+^ and CD8^+^ t cells detection

**Figure 7.**
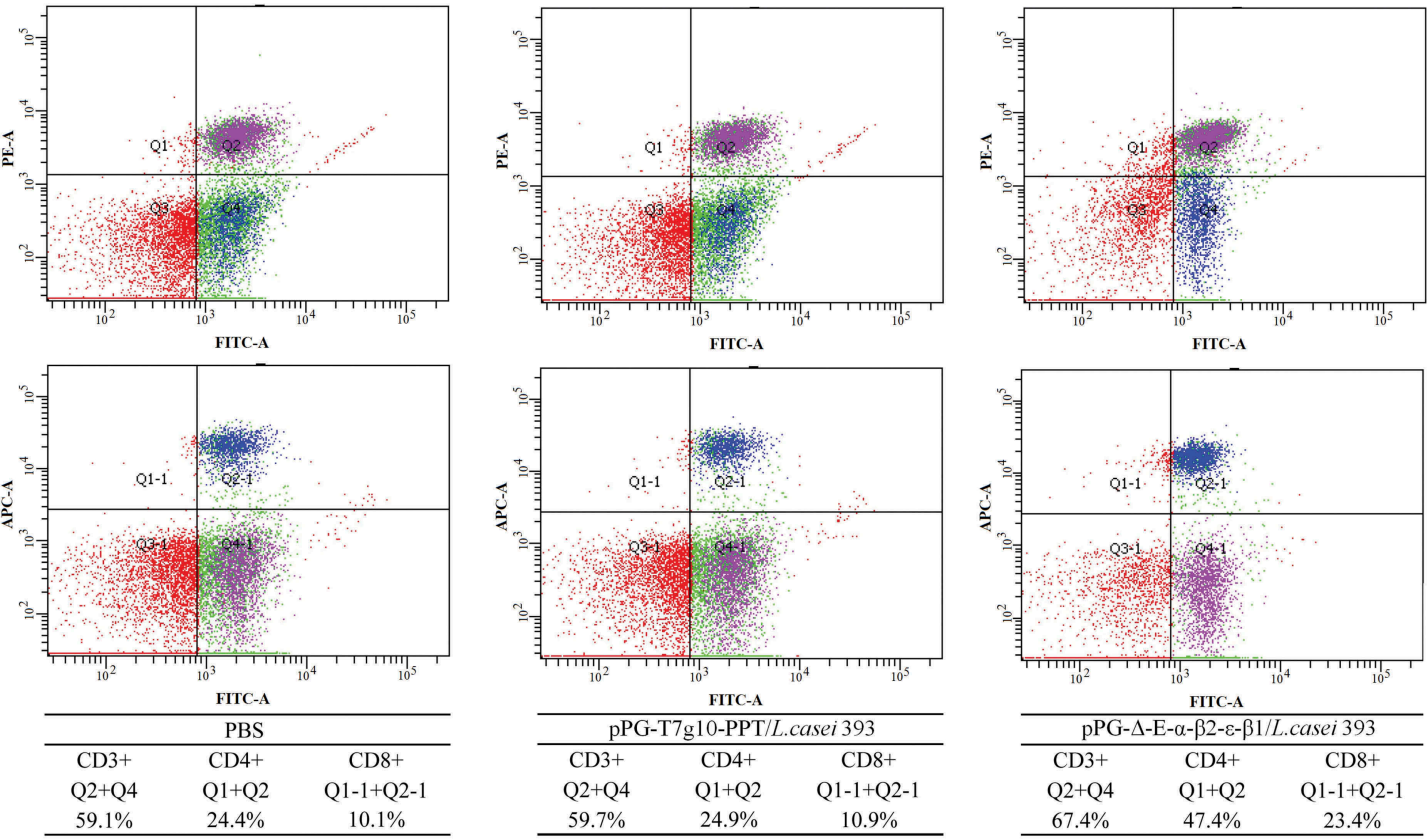
The percentage of CD4+ and CD8 + T cells was determined by flow cytometry. On day 35 after the primary immunization, the spleen lymphocytes of mice in the pPG-Δ-E-α-β2-ϵ-β1/*L. casei* 393, pPG-T7g10-PPT/*L. casei* 393, and PBS groups were respectively isolated, and then the percentage of CD4^+^ and CD8^+^ T cells was analyzed by flow cytometry. The results are presented as mean ± SD in each group.

Spleen lymphocytes were, respectively, collected from the mice orally administrated with pPG-Δ-E-α-β2-ϵ-β1/*L. casei* 393, pPG-T7g10-PPT/*L. casei* 393, and PBS on day 35 after the primary immunization, and then the percentage of CD3^+^, CD4^+^, and CD8^+^ T cells were analyzed by flow cytometry. As shown in Figure 7, the percentages of CD3^+^, CD4^+^, and CD8^+^ T cells in spleen lymphocytes obtained from the pPG-Δ-E-α-β2-ϵ-β1/*L. casei* 393 group was significantly increased, compared to the pPG-T7g10-PPT/*L. casei* 393 or PBS group, but there were no significant differences between the pPG-T7g10-PPT/*L. casei* 393 and PBS groups.

### Lymphoproliferation

**Figure 8.**
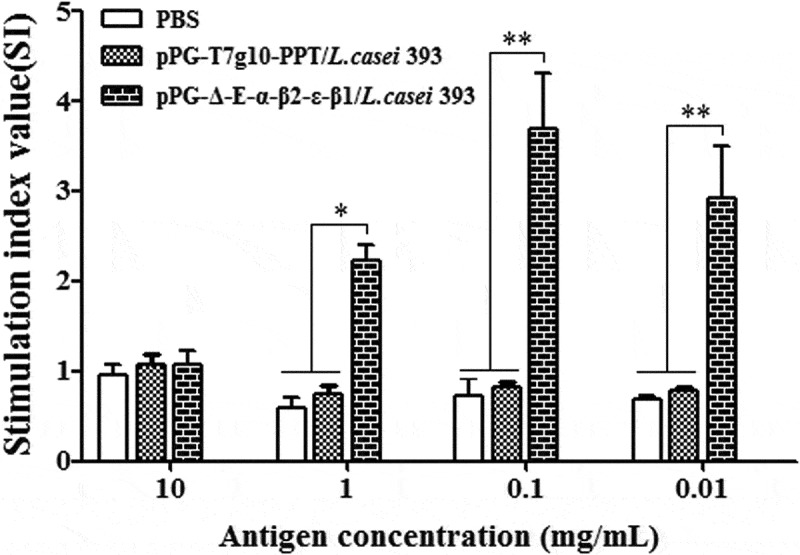
Lymphoproliferation. Spleen lymphocytes of mice in the pPG-T7g10-PPT/*L. casei* 393, pPG-Δ-E-α-β2-ϵ-β1/*L. casei* 393, and PBS groups were collected on day 42 after the primary immunization and were restimulated with the *C. perfringens* α-β2-ϵ-β1 fusion protein, and then lymphoproliferation was analyzed by the MTT assay. The results are presented as mean ± SD of each group.

Spleen lymphocytes of mice orally vaccinated with pPG-Δ-E-α-β2-ϵ-β1/*L. casei* 393, pPG-T7g10-PPT/*L. casei* 393, and PBS was respectively prepared on day 42 after the primary immunization and were restimulated with the *C. perfringens* α-β2-ϵ-β1 fusion protein, followed by lymphoproliferation detection using the MTT assay. The result showed that significant lymphocyte proliferation was observed in immunized mice with pPG-Δ-E-α-β2-ϵ-β1/*L. casei* 393, but not in the pPG-T7g10-PPT/*L. casei* 393 or PBS groups (Figure 8).

### Efficacy of recombinant lactobacilli on immune protection

**Figure 9.**
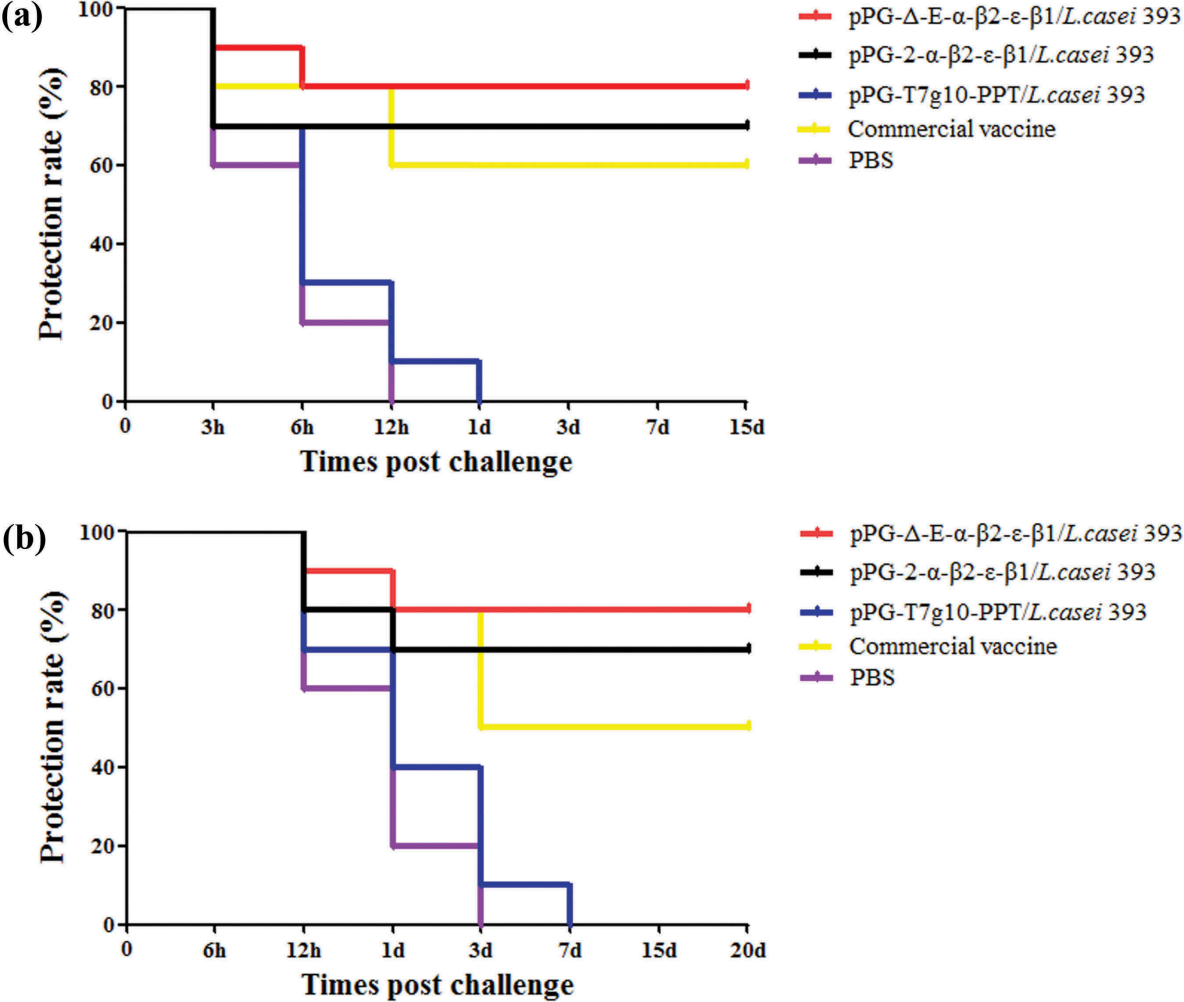
Immune protection efficacy of recombinant lactobacillus. (a) Protection rate for immunized mice in each group against mixture of *C. perfringens* type A, type C, and type D crude toxins. (b) Protection rate for immunized mice in each group against mixture of *C. perfringens* type A, type C, and type D.

On day 35 after the primary immunization, the mice in each group were randomly divided into two subgroups followed by a challenge test. The mice in one subgroup were intramuscularly injected with 1× LD_100_ of a crude toxin mixture produced by *C. perfringens* type A, type C, and type D, and the mice in another subgroup were orally challenged with 2 × 10^9^ CFU of a mixture of *C. perfringens* type A, type C, and type D. The results showed that the survival rates of immunized mice with the pPG-Δ-E-α-β2-ϵ-β1/*L. casei* 393, the pPG-2-α-β2-ϵ-β1/*L. casei* 393, and the commercially available inactivated *C. perfringens* vaccine in the intramuscular injection challenge groups were 80%, 70%, and 60% (Figure 9(a)), and in the oral administration challenge groups were and 80%, 70%, and 50% (Figure 9(b)), respectively, indicating the constitutive recombinant pPG-Δ-E-α-β2-ϵ-β1/*L. casei* 393 was more efficacious in providing immune protection.

### Histopathological analysis

**Figure 10.**
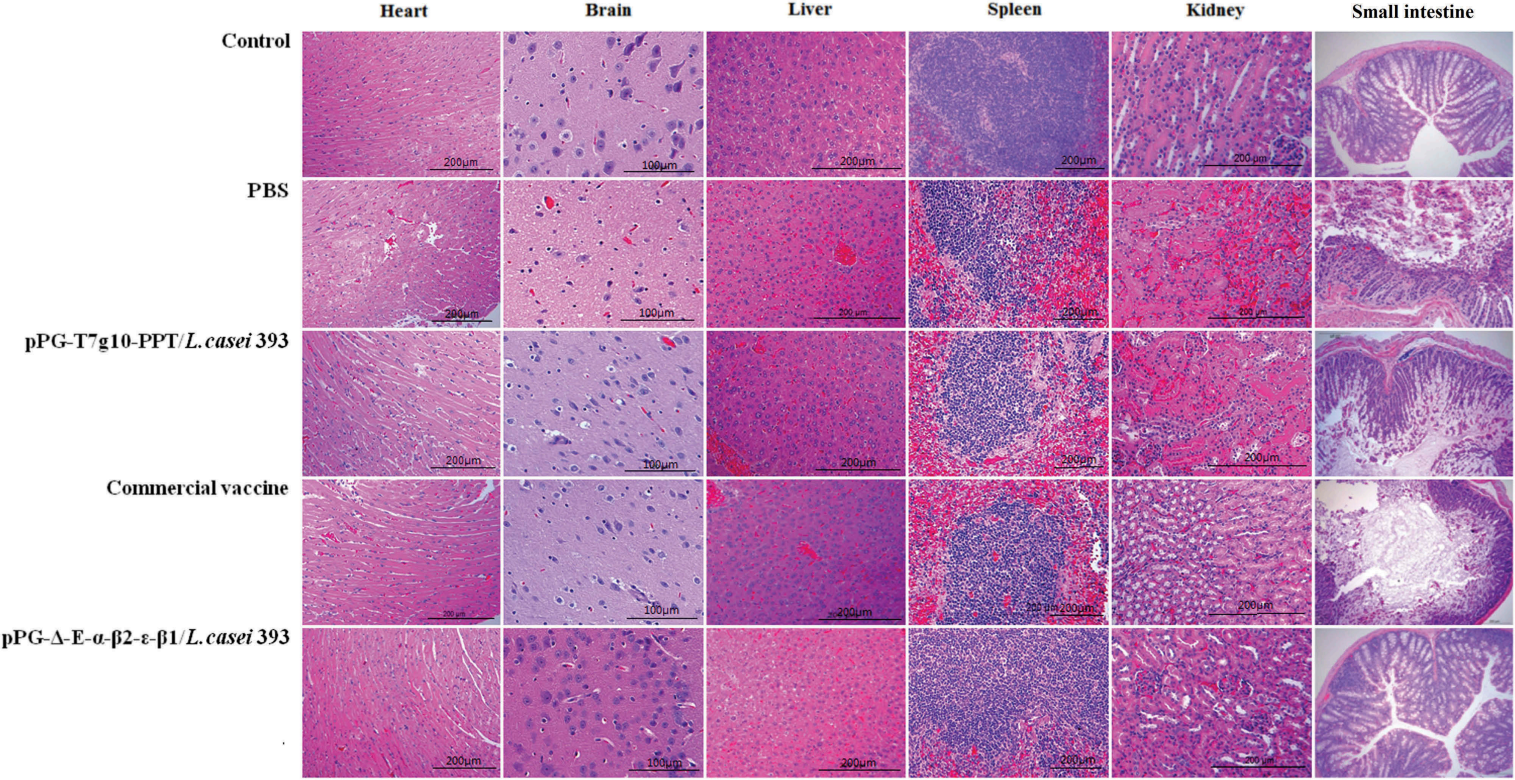
Histopathological changes of immunized mice post-challenge. After challenged with *C. perfringens*, histopathological changes were observed in the intestine, kidney, liver, spleen, heart, and brain of immunized mice with the pPG-Δ-E-α-β2-ϵ-β1/*L. casei* 393, pPG-T7g10-PPT/*L. casei* 393, commercially available vaccine, and PBS. Sections were stained with hematoxylin and eosin, and photographed at 40× magnification.

After being challenged with *C. perfringens*, the liver, intestine, kidney, spleen, heart, and brain of mice in each group were isolated for histopathological observation. As shown in Figure 10, compared to the mice in the normal PBS group (control group without being challenged), there were no abnormal histopathological changes observed in the heart, spleen, intestine, and brain of the mice orally immunized with the recombinant pPG-Δ-E-α-β2-ϵ-β1/*L. casei* 393; slight congestion and hemorrhage changes were found in liver and kidney of the mice in the pPG-Δ-E-α-β2-ϵ-β1/*L. casei* 393 group and the commercial *C. perfringens* vaccine group. The most obvious histopathological changes in the intestine of the mice in the commercial vaccine group, pPG-T7g10-PPT/*L. casei* 393 group, and PBS group was observed, including severe disruption of the intestinal structural integrity, intestinal epithelial necrosis, and shortening of the villi. Moreover, hyperemia, hemorrhages, tubular epithelial cell degeneration/necrosis, and inflammatory cell infiltration were obvious in the histological sections of the kidney and liver of the mice in the pPG-T7g10-PPT/*L. casei* 393 and PBS groups.

## Discussion

*C. perfringens* is a classical opportunistic pathogen in the gastrointestinal tract of livestock and poultry, causing a wide range of diseases such as enterotoxemia, dysentery, and hemorrhagic enteritis [33,35]. The main pathogenic virulence factors of *C. perfringens* are its exotoxins, which are mainly absorbed through the intestinal tract to cause diseases. Although there are many very successful commercial vaccines (toxoids and bacterins) that have been used for decades for a range of *C. perfringens* diseases of animals, there are a few clostridial diseases of animals for which convention vaccines have not been very effective. Thus, the development of vaccines that can effectively induce sIgA-based protective intestinal mucosal immunity against the toxins is of great importance for the control of *C. perfringens* diseases.

Mucosal vaccination by oral administration is a better vaccine formulation against the toxins of *C. perfringens*, which could induce effective systemic immune responses together with mucosal immunity through the mucosal immune system. However, vaccine antigens can easily be destroyed by gastric juice before reaching the intestine tract, generally resulting in a poor immunization effect. Therefore, an ideal antigen delivery carrier is crucial for developing oral vaccines. The delivery of vaccine antigens via viable vectors not only can stimulate the intestinal immune response, but can also produce specific immune responses against specific antigens that allow the body to achieve full protection. Compared to attenuated *Salmonella* as mucosal antigen delivery vehicles [36–38], lactic acid bacteria (LAB) are more advantageous [20,22,23,30,39]. The long-term use of *L. casei* in the food industry has been demonstrated to be non-pathogenic, and has been designated as “generally recognized as safe”. Currently, *L. casei* is recognized as a promising candidate used for delivering therapeutic proteins or antigen to the mucosal surface as a live carrier, which could effectively induce both mucosal and systemic immunity [39].

In the present study, using *L. casei* as an antigen delivery vector, the EGFP-marked recombinant pPG-Δ-E-α-β2-ϵ-β1/*L. casei* 393 expressing α, β2, ϵ, and β1 toxoids proteins of *C. perfringens* was successfully constructed with a constitutive expression plasmid pPG-T7g10-PPT. Currently, inducible expression systems such as xylose [26,33] and nisin [40] are widely used to construct genetically engineered LAB for protein production or vaccine development. However, inducible recombinant LAB has many disadvantages. Particularly, the heterogonous protein must be induced to express by a specific inducer, which greatly limits the application of genetically engineered LAB as an oral vaccine. In this study, the pPG-T7g10-PPT vector, a constitutive lactobacillus expression system constructed by our group [30,31], was used to construct genetically engineered *L. casei* to express α, β2, ϵ, and β1 toxoids proteins of *C. perfringens*, and our results showed that the fusion protein α-β2-ϵ-β1 could be constitutively expressed by the recombinant pPG-Δ-E-α-β2-ϵ-β1/*L. casei* 393 cultured in MRS broth using raffinose, sucrose, lactose, trehalose, glucose and fructose as carbon sources, respectively, and can be recognized by anti-α/β2/ϵ/β1 toxoid monoclonal antibody, indicating that the recombinant *L. casei* constructed in this study can use different carbon sources to grow and express the protein of interest.

As a live mucosal vaccine, the adherence and colonization ability in the intestinal tract is important and desirable. Before oral immunization, we investigated the colonization potentiality of the recombinant pPG-Δ-E-α-β2-ϵ-β1/*L. casei* 393, and the results showed that the pPG-Δ-E-α-β2-ϵ-β1/*L. casei* 393 could survive and adhere to the duodenum, jejunum, ileum, and colon of mice. Although the colonization rate of the recombinant lactobacilli was gradually decreased due to the metabolism of mice and the effects of the digestive tract environments, the CFDA-SE-labeled recombinant lactobacilli in the intestinal tract still remained detectable levels as analyzed by flow cytometry on day 15 after the oral administration.

An effective mucosal vaccine should effectively induce intestinal mucosal and systemic immune responses. Of which, sIgA is the predominant antibody at the mucosal surface, which plays a key role in host protection against pathogenic infections. Thus, we evaluated the immunogenicity in mice of the recombinant pPG-Δ-E-α-β2-ϵ-β1/*L. casei* 393 via oral immunization. Our data demonstrated that the recombinant pPG-Δ-E-α-β2-ϵ-β1/*L. casei* 393 constructed in this study displayed a better immunogenicity, and significant levels (*p*< 0.01) of anti-α/β2/ϵ/β1 toxoid sIgA-based mucosal immune responses and anti-α/β2/ϵ/β1 toxoid IgG-based systemic immune responses were effectively elicited in mice orally immunized with the pPG-Δ-E-α-β2-ϵ-β1/*L. casei* 393 after the second booster, compared to the pPG-T7g10-PPT/*L. casei* 393 or PBS groups. Expectedly, significant levels (*p*< 0.01) of IgG and IgA antibodies could be elicited by the constitutive recombinant pPG-Δ-E-α-β2-ϵ-β1/*L. casei* 393 compared with the xylose-inducible expression recombinant pPG-2-α-β2-ϵ-β1/*L. casei* 393 after the second booster.

Moreover, there was a positive correlation between lymphocyte proliferation rate and cell immunity; thus, we were able to detect the proliferation of spleen lymphocytes in immunized mice. Our results showed that the recombinant pPG-Δ-E-α-β2-ϵ-β1/*L. casei* 393 can also induce cellular immune responses, and higher levels of Th1-type (IL-2, IL-12, IFN-γ) and Th2-type (IL-4, IL-10, IL-17) cytokines were elicited in mice orally immunized with the pPG-Δ-E-α-β2-ϵ-β1/*L. casei* 393 than those in the control groups. CD4^+^ and CD8^+^ T cells are important effector cells for cell-mediated immunity. In this study, the spleen lymphocytes of mice in each group were isolated after immunization, and the percentage of CD4^+^ and CD8^+^ T cells was determined by flow cytometry. The results showed that the percentages of CD4^+^ and CD8^+^ T cells in splenocytes of mice from the recombinant pPG-Δ-E-α-β2-ϵ-β1/*L. casei* 393 group were significantly increased, indicating that the immunization with recombinant lactobacillus promoted the proliferation of CD4^+^ and CD8^+^ T lymphocytes. These results demonstrated that oral vaccination with the pPG-Δ-E-α-β2-ϵ-β1/*L. casei* 393 can induce potent mucosal, cellular, and humoral immune responses. Moreover, using mouse as animal model which is one of the target species of *C. perfringens* infection, the immunoprotection of the recombinant lactobacillus pPG-Δ-E-α-β2-ϵ-β1/*L. casei* 393 against *C. perfringens* toxins and *C. perfringens* were tested, respectively. In parallel, a commercially available inactivated *C. perfringens* vaccine and a xylose-inducible expression recombinant pPG-2-α-β2-ϵ-β1/*L. casei* 393 were used as positive immune control. The protection rates for mice oral immunized with the pPG-Δ-E-α-β2-ϵ-β1/*L. casei* 393, pPG-2-α-β2-ϵ-β1/*L. casei* 393, and commercially available vaccine against crude *C. perfringens* toxins were 80%, 70%, and 60%, and against *C. perfringens* were 80%, 70%, and 50%, respectively, while all the mice in the control groups died, suggesting that the pPG-Δ-E-α-β2-ϵ-β1/*L. casei* 393 is a potential candidate vaccine against the diseases caused by *C. perfringens*, which indicates that constitutive expression lactobacillus system could provide more effective immune protection that inducible expression lactobacillus system. Moreover, the immune protection effect of the probiotic vaccine for other susceptible animals such as rabbits, cattle, and sheep/goat is underway to explore.

Moreover, antibiotic resistance genes such as erythromycin, kanamycin, and chloramphenicol are often used as markers to screen for genetically engineered LAB [26,33,41,42]. Although the use of antibiotic resistance as a screening marker is convenient for screening recombinant lactobacilli, there are potential biosafety risks with the application of antibiotic-resistant recombinant strains in practice, such as the dissemination of antibiotic resistance genes. Therefore, to develop non-antibiotic resistant genetically engineered LAB is of great significance. In this study, the enhanced green fluorescent protein (EGFP) was used as screening marker, and the EGFP-marked pPG-Δ-E-α-β2-ϵ-β1/*L. casei* 393 was successfully developed by a flow cytometry-based screen. Prior to immunization with the probiotic vaccine, we had evaluated the segregational stability and structural stability of the recombinant strain pPG-Δ-E-α-β2-ϵ-β1/*L. casei* 393 during serial subcultures until 50 generations, in the absence of selection pressure, and results showed that the recombinant strain had good stability properties (data not shown). However, before releasing the probiotic vaccine as commercial use in practice, there are more evaluations that need us to perform.

In conclusion, an EGFP-marked genetically engineered lactobacillus strain constitutively expressing *C. perfringens* α, ϵ, β1, and β2 toxoids were developed in this study, and its immunogenicity as an oral probiotic vaccine was investigated. Our results showed that the pPG-Δ-E-α-β2-ϵ-β1/*L. casei* 393 could effectively induce mucosal, cellular, and humoral immune responses via oral administration and provide more effective protection against *C. perfringens* toxins and *C. perfringens* infections than inducible expression lactobacillus system, suggesting a promising candidate for the development of oral vaccine against *C. perfringens*. Actually, we will hardly have a problem with all these toxinotypes in the field. Therefore, it would be of great value to construct and test the separate toxinotypes.
